# Suitability of specific soft tissue swabs for the forensic identification of highly decomposed bodies

**DOI:** 10.1007/s00414-021-02601-3

**Published:** 2021-04-20

**Authors:** Katharina Helm, Christian Matzenauer, Franz Neuhuber, Fabio Monticelli, Harald Meyer, Stefan Pittner, Walther Gotsmy

**Affiliations:** Department of Forensic Medicine and Forensic Neuropsychiatry, University of Salzburg, Salzburg, Austria

**Keywords:** Forensic identification, Decomposition, Soft tissues, DNA profiling, STR marker

## Abstract

When decomposition of a recovered body is fairly advanced, identification based on common morphologic features is often impossible. In these cases, short tandem repeat (STR) marker genotyping has established itself as a convenient and reliable alternative. However, at very progressed stages of decomposition, postmortem tissue putrefaction processes can decrease DNA yields considerably. Hence, not all types of tissue are equally suitable for successful STR marker-based postmortem identification.

Bone or dental material is often analysed in corpses with advanced decompositional changes. However, processing of these materials is very elaborate and time and resource consuming. We have therefore focused on the suitableness of various types of soft tissue swabs, where DNA extraction is easier and faster. By sampling 28 bodies at various stages of decomposition, we evaluated the suitability of different tissues for genotyping at varying degrees of physical decay. This was achieved by a systematic classification of the sampled bodies by morphological scoring and subsequent analysis of multiple tissue swabs of the aortic wall, urinary bladder wall, brain, liver, oral mucosa and skeletal muscle.

In summary, we found variable degrees of suitability of different types of soft tissue swabs for DNA-based identification. Swabs of the aortic wall, the urinary bladder wall and brain tissue yielded the best results — in descending order — even at advanced levels of decay.

**Supplementary Information:**

The online version contains supplementary material available at 10.1007/s00414-021-02601-3.

## Introduction

A reliable and rapid forensic identification after the recovery of a human body is a crucial and morally obligatory task in many respects. However, when decomposition processes are already fairly advanced and common morphological identifying features are no longer reliable, alternative methods are required. Here, in most cases even with highly decomposed bodies, the identity of a human corpse can still be established by DNA profiling — more specifically by short tandem repeat (STR) marker profiling [1–4]. Still, DNA yields decrease with progressing decay due to microorganism activity and autolytic processes such as DNAse activity, hydrolysis and oxidation [5]. Some kinds of tissue are known to be less susceptible to these processes than others. Therefore, the choice of tissue is relevant to gain suitable material for achieving the task of forensic DNA profiling.

When bodies are highly decomposed or even skeletonised or mummified, DNA extracted from dental or bone material is a valuable approach for a successful forensic identification [6–11]. However, the process of DNA extraction from this tissue type is very elaborate and time and resource consuming. DNA extraction from soft tissues, on the other hand, is faster and less extensive. Another valuable source for DNA typing of highly decomposed bodies is human fingernails or toenails [12–14]. Here, DNA extraction is similarly unsophisticated. But with this type of tissue, contamination can be an issue, especially in criminal cases with rape scenarios before death. Moreover, at advanced stages of decomposition, fingernails and toenails can get detached from the body and can possibly not be ascribed with undisputable certainty to a recovered body.

Several studies centre on the suitability of different human bone types or specific dental tissue for a successful forensic identification [15–21]. Although the success of STR typing, dependent on the progress of decay, has generally been investigated in soft tissue [2, 22–28], literature directly comparing different soft tissue types at various stages of decomposition for forensic identification purposes is sparse [29, 30]. Therefore, we focused on a selection of swabs from soft tissues, such as the aortic wall, the urinary bladder wall, brain tissue, liver tissue and skeletal muscle tissue to gain more information about the reliability of those tissues even at more advanced stages of decomposition. Tissue selection was based on experiences gained in our routine casework. Aorta and urinary bladder are well-protected hollow organs, easily swabbed. The brain, liver and muscle were selected as they originate from different body regions and underly very different internal decomposition influences. Oral swabs were collected as a reference. Swabs were chosen as sampling method, as this is a straightforward, common, valid standard method in most DNA laboratories. Other than that, this sampling method is fast and easily applicable during autopsies. Furthermore, DNA extraction from swabs is less elaborate than DNA extraction from original tissue samples. There are several environmental factors that can influence decomposition of the whole human body as one entity but also degradation of single organs and tissue types can vary based on changed external influences. Those environmental factors include clothing, temperature, humidity, insect- or microorganism-activity, scavenging and body composition before death [31–36]. To achieve a better comparability between cases and sampled tissue types for this study, we exclusively selected cases of highly decomposed bodies that were discovered indoors. Another factor to achieve a better comparability and to ensure improved evaluation of these tissue types is a categorization of the state of decomposition of the bodies analysed in this study. To additionally implement this factor into our investigation, two separate scoring methods based on morphological features of ongoing decay were used: The Total Decomposition Score (TDS) and the Total Body Score (TBS) [37, 38]. The first scoring method by Gelderman et. al provides a guide to calculate a “Total Decomposition Score” (TDS). This score represents the sum of three separately evaluated regional scores: “facial decomposition score” (FDS), “body decomposition score” (BDS) and the “limbs decomposition score” (LDS). These have specific point-values depending on morphological features in relation to the decomposition process, ranging from 3 (fresh) to 18 (complete skeletonization) possible points [37]. The second scoring method by Megyesi et al. has similar features. In this case, the values representing the separately evaluated regional scores add up to the Total Body Score (TBS) ranging from 3 (fresh) to 35 (dry bone) points, also depending on the degree of decomposition [38]. The aim of this study was to establish whether a swab of a certain type of soft tissue is still suitable for a valid forensic identification even at advanced degrees of putrefaction.

## Materials and methods

### Sampling and morphological scoring

In this study, samples of in total 28 bodies with a higher degree of decomposition were taken (Tables 1 and 2). Those bodies were sampled and analysed between April 2019 and February 2020 at the Institute for Forensic Medicine in Salzburg, Austria. We chose a variety of soft tissues for sampling and subsequent DNA profiling. Sampling was mostly of an invasive nature during the autopsy of our cases. Two swabs each (one as backup) of the aortic wall, the urinary bladder wall, brain tissue, liver tissue and skeletal muscle tissue were taken as well as oral swabs. The muscle samples were all taken from the *Musculus vastus lateralis*. In this manner, a set of 336 samples was produced and further processed. To accomplish a systematic classification of the decomposition state of all analysed bodies, the degree of decomposition was evaluated with the two specific scoring methods based on morphologic features TDS and TBS.

**Table 1:** Overview of all 28 individuals included in this study and respective grades of decomposition. Both scoring systems score decomposition of three body regions by assigning point values from a minimum of 3/3 (fresh) to a maximum of 18/35 (complete skeletonization/dry bone) in TDS/TBS

Case No	Size (cm)	Weight (kg)	Gender	TDS [37]	TBS [38]
1	185	60	F	9	14
2	174	75	M	12	20
3	182	110	M	9	12
4	168	51	M	12	17
5	188	90	M	11	18
6	144	30	F	12	18
7	173	75	M	9	15
8	175	80	M	12	19
9	169	75,8	M	8	13
10	176	42	M	10	15
11	170	75	M	11	20
12	178	75	M	10	15
13	153	72	F	13	21
14	154	50	F	10	17
15	174	75	M	11	16
16	167	65	M	12	16
17	175	40	M	12	22
18	179	80	M	10	16
19	178	80	M	8	9
20	187	95	M	10	16
21	165	45	F	13	25
22	161	53	F	12	22
23	190	90	M	9	14
24	163	42	F	10	16
25	153	26	M	12	18
26	172	71	M	6	6
27	177	68	M	9	18
28	160	45	F	12	25

**Table 2:** Profile completeness of all 28 cases of highly decomposed bodies included in the study. Average success rates were calculated for each soft tissue type. (*c*, contaminated)

Case ID		TDS		TBS		Aorta	Urinary bladder		Brain		Liver	Oral swab	Muscle
1		9		14		100	100		100		100	100	100
2		12		20		100	100		100		100	80	100
3		9		12		100	100		100		100	100	100
4		12		17		100	100		100		100	94	100
5		11		18		100	100		100		100	97	100
6		12		18		100	86/c		97		82/c	100/c	31
7		9		15		100	100		100		80	100	21
8		12		19		100	100		100		100	78	100
9		8		13		100	100		100		100	100	100
10		10		15		100	100		100		100	100	100
11		11		20		100	100		43		100	0	100
12		10		15		100	100		100		100	100	100
13		13		21		100	100		100		100	37	100
14		10		17		100	100		100		100	41	99
15		11		16		100	100		100		100	49	100
16		12		16		86	97		59		90	99	81
17		12		22		100	100		88		100	25	100
18		10		16		100	100		93		100	22	26
19		8		9		100	100		100		100	100	100
20		10		16		100	97		100		91	90	100
21		13		25		100	100		100		94	0	97
22		12		22		100	100		100		74	41	100
23		9		14		100	100		95		100	100	100
24		10		16		100	100		100		100	100	100
25		12		18		100	100		100		100	31	100
26		6		6		100	100		100		100	100	100
27		9		18		100	100		100		100	100/c	100
28		12		25		100	100		100		100	94	60
**28 cases**		**6–13**		**6–25**		**27**	**25**		**22**		**22**	**12**	**21**
	**99.5**		**99.3**		**95.5**		**96.8**	**74.2**		**89.8**			

### DNA extraction

DNA extraction of all samples was performed with the GEN-IAL ®—First DNA all-tissue DNA Kit according to the manufacturer’s instructions [39].

### DNA quantification and amplification

DNA extracts were quantified using the Quantifiler™ Trio DNA Quantification Kit (Thermo Fisher Scientific Corporation) and a 7500 Real-Time PCR System for Human Identification (Thermo Fisher Scientific Corporation). Subsequently, PCR amplification was performed on a Gene Amp PCR Systems 9700 system using the AmpFlSTR™ NGM SElect™ PCR Amplification Kit (Thermo Fisher Scientific Corporation) at 29 cycles with max 1 ng of DNA input based on the DNA yields determined in the quantification step. For all samples, two individual PCR runs were performed as internal quality control and to eliminate possible methodological errors.

### Capillary electrophoresis and data analysis

For fragment separation, a mixture of 2–5 µl amplified PCR product, 23.5 µl HiDiTM formamide (Thermo Fisher Scientific Corporation) and 1.5 µl GeneScanTM 600 LIZ ® dye Size Standard v2.0 (Thermo Fisher Scientific Corporation) was injected in an ABI 3500 Genetic Analyzer (Thermo Fisher Scientific Corporation). The obtained DNA STR marker profiles were then analysed, and profile quality was evaluated (GeneMapper ® ID-X software Version 1.4, Thermo Fisher Scientific Corporation). Here, the minimum signal strength analysed was 50 relative fluorescent units (RFU). Profile completeness was calculated as the percentage of observed alleles out of a full STR marker profile. A full profile is represented by 68 alleles in total: 17 STR marker systems with two heterozygous or homozygous single alleles per system in two PCR approaches. Average success rates per tissue type were then accessorily calculated.

### DNA re-extraction and re-amplification

Of all samples that showed very low DNA yields or signs of contamination, the backup swab was additionally analysed (*n* = 22) in the same manner as the first swab. If the second swab was also of poor quality, or samples still showed signs of contamination, they were labelled accordingly and excluded from further evaluation.

### Secondary amplification and RFU analysis

In a next step, all samples that provided a higher DNA concentration yield than 0.1 ng/µl and a full 17 locus DNA profile (34 alleles out of 17 STR Marker systems in a PCR double approach adding up to 68 alleles) were further processed in the following manner: Samples were diluted with DNA-free water to achieve a standardised concentration of 0.1 ng/µl. After that, another amplification step was performed in the same setup as mentioned above with 5 µl DNA (= 500 pg) input. For a subsequent fragment separation step, 2 µl of the amplified products was used. To obtain an additional parameter for evaluation of the obtained DNA profiles of all tissue kinds, DNA degradation was evaluated by RFU analysis. The RFU per allele was calculated as the average of the heterozygous peak heights or the homozygous peak height divided by two. The max:min ratio was then calculated by dividing the value of the highest RFU per allele of all loci within an electropherogram of a sample with the lowest RFU per allele of all loci in the same electropherogram [17]. This was performed with both PCR approaches per sample and then an average was calculated. Statistical calculations were performed with Microsoft Excel 2019 (Version 16.33).

## Results

**Fig. 1:**
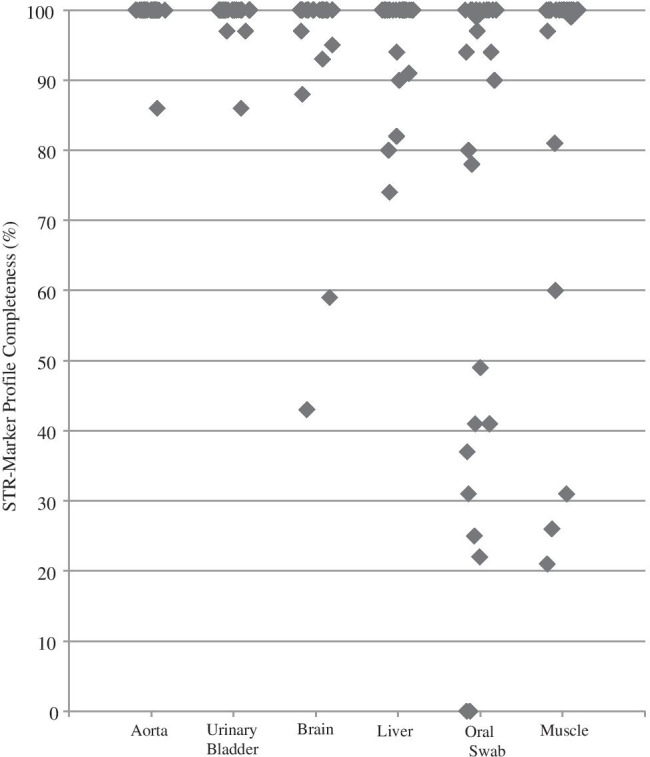
Schematic presentation of the STR typing results from six types of soft tissues after amplification with the AmpFlSTR™ NGM SElect™ PCR Amplification kit (Thermo Fisher Scientific Corporation). Shown are the profile qualities of each tissue type (100% ≙ 68 alleles)

**Fig. 2:**
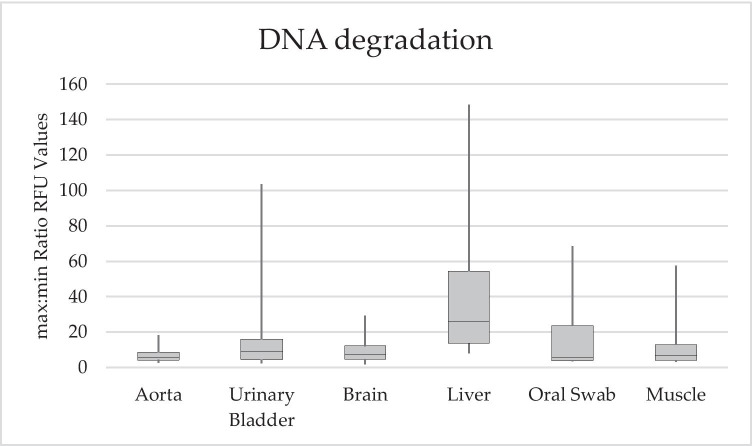
Box whisker plot showing the Degradation Scores of all uncontaminated samples with a DNA concentration above 0.1 ng/µl, calculated separately for each soft tissue

In general, success rates were rather high regarding STR marker profile completeness (see Table 2 and Fig. 1). The swab of the aortic wall showed the best results with a 99.5% average success rate in STR marker profile completeness. Sampling the urinary bladder led to 25 out of 28 samples with full profiles (average success rate of 99.3%). The brain and liver swabs showed quite similar results with 95.5% and 96.8%, followed by the muscle samples with 89.8%. The oral swabs did provide the least successful profile completeness rate with only 74.2%. As sampling the aorta provided a full profile in 27 out of 28 cases, this is not surprising. To gain information about the degradation of the analysed samples, a max:min ratio of the RFU values was calculated. The higher the max:min ratio, the higher the difference between peak heights within an electropherogram of one sample, indicating a “ski-slope-effect” and therefore higher degradation. This effect is caused by the observation that in degraded DNA, signal intensities decrease with increasing fragment length [40]. To ensure a better comparability between samples, only samples with a concentration above 0.1 ng/µl were further processed with standardised conditions. Here, as shown in Fig. 2 and Table 3, the liver samples showed the highest degradation compared to other samples and a quite high standard deviation. The degradation values calculated for the aorta samples are the lowest compared to the other soft tissue samples. Our samples were evaluated with morphological scores ranging from 6 to 13 (TDS) and 6 to 25 (TBS) (see Table 1, Supplementary Table S2). Up to a TDS of 9 and a TBS of 14, all sampled tissues do still provide a full quality STR marker profile. All sampled cases except one with a TDS below 10 and a TBS value below 16 do provide a profile completeness of more than 95%. If the TDS score increases above 11 and the TBS above 18 at least one of the sampled soft tissue types does not provide a 100% quality profile. The oral swab does not provide a 100% profile completeness in all but one sample with a TDS above 10 and a TBS higher than 16. In 27 out of 28 cases, the aorta samples did provide a full profile even at the highest TDS and TBS scores analysed. The same picture applies for the urinary bladder wall swabs with a profile completeness of 95% in all samples but one.

**Table 3:** Degradation levels of all samples (with a DNA concentration of > 0.1 ng/µl and a full profile), calculated as max:min ratio of the RFU values. Higher values indicate increased DNA degradation [17]. *x*, concentration < 0.1 ng/µl; *IP*, incomplete profile; *c*, contamination

Case ID	TDS	TBS	Aorta	Urinary bladder	Brain	Liver	Oral swab	Muscle
1	9	14	8.8	3.5	x	10.5	5.2	18.6
2	12	20	8.0	x	10.3	c	x	6.9
3	9	12	2.5	5.9	1.7	15.8	3.7	3.5
4	12	17	18.2	6.5	12	19.5	x	14.1
5	11	18	12	9	8.7	IP	x	37.5
6	12	18	x	x	x	x	x	x
7	9	15	13.3	4.4	5.5	x	x	x
8	12	19	5.5	10.3	6.3	13.6	x	8.9
9	8	13	6.0	3.6	2.9	56.7	3.8	4.0
10	10	15	6.0	7.8	2.8	7.9	5.8	7.2
11	11	20	3.0	16.6	x	11.7	x	9.3
12	10	15	11.2	5.6	12.5	48.4	68.5	3.4
13	13	21	3.5	28.4	9.0	20.4	x	x
14	10	17	10.2	103.5	5.8	148.2	x	IP
15	11	16	4.4	15.9	6.0	74.7	x	6.8
16	12	16	x	IP	x	x	29.5	IP
17	12	22	4.1	4.1	IP	IP	x	x
18	10	16	4.7	13.4	x	61.6	x	x
19	8	9	2.8	2.4	2.9	26.0	3.4	3.2
20	10	16	8.1	2.2	5.2	IP	x	3.3
21	13	25	x	14.9	x	x	x	57.5
22	12	22	x	39.8	12.5	IP	x	x
23	9	14	7.0	4.4	29.2	9.7	4.4	4.2
24	10	16	5.0	18.5	28.7	54.8	29.2	6.1
25	12	18	x	25.8	9.6	27.14	x	x
26	6	6	3.7	4.9	3.2	13.5	6.7	6.4
27	9	18	x	10	19.2	54.0	c	49.7
28	12	25	4.4	14.3	x	36.0	x	x

## Discussion

To ensure a valid and rapid forensic identification, the selection of tissue type for a DNA analysis seems to be an important factor. With progressing decomposition in a human body, choices are increasingly limited due to tissue putrefaction and advanced DNA degradation processes. Bone and dental material are recommendable and widely used as the tissue of choice and at some point, during these processes when skeletonization or mummification is already fairly advanced, those hard tissue types are the only tissues available for sampling. However, if still possible, the sampling of soft tissues for forensic identification through DNA typing is by far less elaborate. In the present study, six types of soft tissue swabs sampled from highly decomposed bodies were examined: aortic wall, urinary bladder wall, brain, liver, skeletal muscle and oral mucosa. To achieve this, profile quality and DNA degradation from all tissue swabs were analysed and a possible correlation with two different degradation scores based on morphologic features was evaluated.

For amplification and subsequent generation of STR marker profiles, solely the AmpFlSTR™ NGM SElect™ PCR Amplification Kit was used as we have positive experiences with using this kit for the identification of heavily putrefied bodies. For the application in routine forensic laboratory case work, it is recommendable though to use a combination of kits (e.g. in combination with the Power Plex® ESI 17 Pro System).

To answer the research question of this study, it is quite important to “quantify” and categorise the state of decomposition of the recovered bodies. Both scoring methods, the TDS and the TBS, are based on specific morphologic phenomena which are linked to certain stages of decomposition and both showed similar results for each body analysed in this study. Notably, organ or tissue decomposition can differ from overall morphological decomposition scoring. However, in regard of the lack of decomposition scoring methods for these units, a global decomposition score comprising three body regions was considered best practice.

The results of the current study show that the aortic swabs provided the best profile completeness at all analysed levels of decay with a 99.5% success rate of achieving a full STR marker profile with a sample size of 28 analysed bodies. Sato et al. found that the aorta is one of the most useful samples for forensic identification after analysing 47 bodies with a variety of causes of death, including drowning and burning [26, 41]. These findings are supported by the results of a study of Shintani-Ishida analysing blood vessels in general as a promising source for DNA typing [42]. Thus, the aortic wall seems to be a quite resilient and dependable type of tissue for sampling even under severe conditions. Another reliable tissue under challenging forensic conditions is the urinary bladder. It is the tissue of choice for the identification of burnt bodies due to its anatomically protected situation within the body [23]. As antemortem bacterial colonization in this area is highly suppressed by innate immune system activity, also postmortem decay is delayed until very advanced body degradation. This could explain why the urinary bladder wall swabs showed the second-best results regarding profile completeness and low degradation values compared to other tissues analysed in this study.

Brain tissue provided a 95.5% average success rate in this study and good-quality DNA even at higher TDS/TBS rates. This could be explained by its enclosed location in the skull, preserving it from environmental factors such as bacterial or insect colonization for a longer time period than abdominal or thoracic organs. Other than that, the brain mass is not only protected by the *ossa cranii* but also by the *dura mater*. Motani et al. showed in their study that the *dura mater* itself is still a good source for DNA profiling even at higher degrees of decomposition [25]. Likewise, Ludes et al. and Pooniya et al. denominated brain tissue as a good source for DNA fingerprinting [30, 43].

Enhanced postmortal enzymatic and bacterial activity can be seen in the area of another organ analysed in this study: the liver. Due to these DNA degrading processes, this type of tissue is not highly dependable for DNA typing. Our results showed only in 22 out of 28 cases a full STR marker profile. In a study by Hoff-Olsen et al., incomplete profiles could be seen in more than half of their investigated cases [2]. Insect activity and especially maggot infestation are another factor that can severely influence soft tissue quality for DNA profiling. As postmortem, maggots enter the body primarily through natural body openings like the mouth, nose and the eyes, and putrefaction processes can be accelerated in these areas. Antemortem, a buccal swab is standard procedure for a fast and valid identification of a person. But during decomposition, the amount of liquid decompositional products is quite high which can make sampling difficult. Together with the aforementioned insect activity, this leads to poor-quality DNA extraction of oral swabs of highly decomposed bodies.

The DNA profiling results for skeletal muscle tissue are oscillating rather much compared to other soft tissue types. This phenomenon could also be observed in other studies [24, 27].

When TDS/TBS values are correlated to profile completeness, our data suggest that all investigated tissues are viable sources for DNA sampling up to a TDS/TBS of 9/14 (e.g. grey to green discoloration of the skin, early bloating stages) and respectively 95% profile completeness up to 10/16 (e.g. purging of decompositional fluids, brown to black discoloration of limbs). All cases with higher scores failed to yield complete profiles from oral swabs, and this source should thus be avoided. Aorta and urinary bladder performed extremely well, with only a single case yielding under 95% profile completeness respectively. Notably, these were not the cases with the highest overall decomposition scores (12/16 and 12/18, e.g. brown discoloration of the head and neck, sagging of flesh, caving in of the abdominal cavity), suggesting additional influences. Muscle, the brain and liver also performed surprisingly well, with average success rates of around 90–95%. Although there was a trend observed that cases with higher TDS/TBS values produced less conclusive outcome, even some with very high values (e.g. 13/21, 12/25, e.g. bone exposure, (partial) mummification) yielded complete profiles in these tissues.

After sampling hard tissues, like bone or dental material, a good comparability of DNA yields can be achieved by using similar amounts of e.g. bone powder for subsequent DNA extraction. With soft tissue samples, it is not as easy to find a good sampling method that satisfies this criterium. The utilization of swabs is a fast and easy standard sampling method for soft tissues in general and for the forensic identification of fresh human bodies. Therefore, we chose this sampling method in our study. However, DNA concentration values after the extraction process cannot be conclusively compared as while swabbing various types of soft tissue in various stages of decay, it is not possible to sample a comparable “amount” of tissue. Therefore, we did not compare DNA yields in this study and “standardised” samples above 0.1 ng/µl for further analysis to ensure a better comparability. Original DNA yields are summarised in Supplementary Table S1.

In general, the application of swabs for sampling soft tissues of highly decomposed bodies proved to be a valid and convenient method. Nevertheless, future research, directly comparing the tissue swabs to direct tissue extraction, could improve understanding of the suitability of tissue swabs.

The decay of bodies in an outdoor environment is influenced by more factors than the decay of bodies degrading indoors, which alters the decomposition rate and consequently the condition of tissue types more intensely [34]. Nevertheless, also indoors, there are still many environmental factors and inter-individual variation that influence degradation processes [44] and this can be seen as a limitation of this study. Increasing the sample size could average out the large number of variables which can affect DNA preservation. Based on our results, we recommend the sampling of the aorta, urinary bladder or brain tissue for the identification of highly decomposed bodies by DNA fingerprinting. However, further research is required in this field and the comparison of these tissue types with other tissues such as cartilage, Achilles tendon, fingernails and toenails and ocular swabs is desirable as those also have been labelled as useful for DNA extraction in cases of putrefied unidentifiable bodies [24, 28, 45].

## Conclusion

In this study, we discovered differences in the suitability of a variety of soft tissue type swabs for the forensic identification of highly decomposed bodies by DNA profiling. Swabs of the aortic wall, the urinary bladder wall and brain tissue showed the most promising results respectively. Swabs of muscle and liver tissue did not provide stable results for viable profiling at varying degrees of decomposition. Oral swabs are highly recommendable for identification purposes antemortem but not postmortem when increased levels of decay are reached.

## Supplementary Information

Below is the link to the electronic supplementary material.

Supplementary file1 (XLSX 15 KB)

## Declarations

All applicable international, national and/or institutional guidelines were followed.
